# Admission Predictors of Mortality in Hospitalized COVID-19 Patients—A Serbian Cohort Study

**DOI:** 10.3390/jcm11206109

**Published:** 2022-10-17

**Authors:** Mina Poskurica, Đorđe Stevanović, Vladimir Zdravković, Ivan Čekerevac, Vojislav Ćupurdija, Nebojša Zdravković, Tomislav Nikolić, Marina Marković, Marina Jovanović, Marija Popović, Katarina Vesić, Ana Azanjac Arsić, Snežana Lazarević, Andra Jevtović, Aleksandar Patrnogić, Marija Anđelković, Marina Petrović

**Affiliations:** 1 Cardiology Clinic, University Clinical Center Kragujevac, 34000 Kragujevac, Serbia; 2 Department of Internal Medicine, Faculty of Medical Sciences, University of Kragujevac, 34000 Kragujevac, Serbia; 3 Pulmonology Clinic, University Clinical Center Kragujevac, 34000 Kragujevac, Serbia; 4 Department of Medical Statistics and Informatics, Faculty of Medical Sciences, University of Kragujevac, 34000 Kragujevac, Serbia; 5 Urology and Nephrology Clinic, University Clinical Center Kragujevac, 34000 Kragujevac, Serbia; 6 Center of Medical Oncology, University Clinical Center Kragujevac, 34000 Kragujevac, Serbia; 7 Gastroenterohepathology Clinic, University Clinical Center Kragujevac, 34000 Kragujevac, Serbia; 8 Neurology Clinic, University Clinical Center Kragujevac, 34000 Kragujevac, Serbia; 9 Department of Neurology, Faculty of Medical Sciences, University of Kragujevac, 34000 Kragujevac, Serbia; 10 Otorhinolaryngology Clinic, University Clinical Center Kragujevac, 34000 Kragujevac, Serbia; 11 Department of Otorhinolaryngology, Faculty of Medical Sciences, University of Kragujevac, 34000 Kragujevac, Serbia; 12 Center for Laboratory Diagnostics, University Clinical Center Kragujevac, 34000 Kragujevac, Serbia; 13 Department of Biochemistry, Faculty of Medical Sciences, University of Kragujevac, 34000 Kragujevac, Serbia

**Keywords:** admission predictors, COVID-19, mortality

## Abstract

Background: Early prediction of COVID-19 patients’ mortality risk may be beneficial in adequate triage and risk assessment. Therefore, we aimed to single out the independent morality predictors of hospitalized COVID-19 patients among parameters available on hospital admission. Methods: An observational, retrospective–prospective cohort study was conducted on 703 consecutive COVID-19 patients hospitalized in the University Clinical Center Kragujevac between September and December 2021. Patients were followed during the hospitalization, and in-hospital mortality was observed as a primary end-point. Within 24 h of admission, patients were sampled for blood gas and laboratory analysis, including complete blood cell count, inflammation biomarkers and other biochemistry, coagulation parameters, and cardiac biomarkers. Socio-demographic and medical history data were obtained using patients’ medical records. Results: The overall prevalence of mortality was 28.4% (*n =* 199). After performing multiple regression analysis on 20 parameters, according to the initial univariate analysis, only four independent variables gave statistically significant contributions to the model: SaO2 < 88.5 % (aOR 3.075), IL-6 *>* 74.6 pg/mL (aOR 2.389), LDH *>* 804.5 U/L (aOR 2.069) and age > 69.5 years (aOR 1.786). The C-index of the predicted probability calculated using this multivariate logistic model was 0.740 (*p <* 0.001). Conclusions: Parameters available on hospital admission can be beneficial in predicting COVID-19 mortality.

## 1. Introduction

COVID-19 is a multisystemic disease, caused by Severe acute respiratory syndrome coronavirus 2 (SARS-CoV-2), with a mortality count of more than 6.4 million people worldwide [1,2]. Although most patients experience mild to moderate clinical course, approximately 20% of patients develop severe forms of the disease associated with respiratory failure, acute respiratory distress syndrome (ARDS), multi-organ damage, and other complications [3]. Despite continuous efforts and research on this topic, the exact mechanisms and the circumstances in which they trigger the disease progression and lethal outcome are not fully understood [2,4]. More than thirty parameters associated with COVID-19 mortality can be found in the literature, including older age, male gender, presence of certain comorbidities, gas exchange impairment, and various laboratory analyses (abnormalities of blood count and leukocyte formula, impaired coagulation status, and elevated biomarkers of inflammation, myocardial, renal, and hepatic impairment) [5,6,7,8,9,10,11,12,13,14,15,16,17,18]. However, the selection of significant predictors, their cut-off, and adjusted odds ratio (aOR) values differ across the literature. These diversities can partially be explained by frequent SARS-CoV-2 mutations, differences in methodological approach and variable selection, heterogeneity of the studied population, and other study variations. In addition, data regarding analyses conducted on admission laboratory findings are limited [9,12,19,20].

To have clearly defined and accessible predictors of COVID-19 severity and mortality, which can be used in the initial risk assessment upon hospital admittance, would be beneficial in a major health system burden caused by the actual pandemic. Since, to our knowledge, such a study was not conducted in Serbia on a large sample, our research aimed to single out mortality predictors among parameters available in the first hours of hospital admission.

## 2. Materials and Methods

### 2.1. Study Population

The study was a part of the “COVID-19 admission PREDICTors of OUTCOME” (COVID-19 PREDICT OUTCOME) registry. An observational, prospective cohort study was conducted on 703 consecutive COVID-19 patients hospitalized in the University Clinical Center Kragujevac between September and December 2021. In-hospital mortality was the primary end-point, and patients were followed during the hospitalization. Inclusion criteria were: (1) adult age (≥18 years old), (2) patient consent to participate in the study, (3) confirmed SARS-CoV-2 infection by RT-PCR or antigen test [3], and (4) definite discharge or COVID-19-related death outcome. General exclusion criteria were: (1) patient refusal to participate in the study or impossibility to obtain a consent form (due to critical/unconscious state), (2) pregnancy and the early postpartum period, (3) initial hospitalization in our Center for non-COVID pathology, (4) terminal stage of malignant disease, (5) lack of complete admission laboratory analysis of interest, (6) continuation of further inpatient treatment in another facility, and (7) transport from other institutions due to a critical state. We note that 1211 patients were hospitalized in our COVID center during the data collection. However, 508 patients were excluded according to exclusion criteria.

### 2.2. Data Collection

Socio-demographic and medical history (regarding comorbidities and outpatient disease course) data were obtained anamnestically and using patients’ electronic medical records (Health Informational System, ComTrade, Kragujevac, Serbia). Within 24 h of admission, patients were sampled for blood gas and laboratory analysis, including complete blood cell count, inflammation biomarkers, and other biochemistry and coagulation parameters, as well as cardiac biomarkers.

### 2.3. Statistical Analysis

Statistical analysis was performed using the SPSS statistical package, version 23.0 (IBM corporation, Armonk, NY, USA). Continuous variables were presented as median with interquartile range (IQR) and categorical data as the absolute and relative frequency. Differences in quantitative data were tested using the Mann–Whitney U test and their relationship using Spearman’s correlation, according to the non-normal distribution assessed by the Kolmogorov–Smirnov test. If applicable, continuous data were further transformed into binary variables, using the accepted reference line or cut-off values given by the receiver operating curve (ROC) analysis, concerning Youden’s index for achieving optimal specificity and sensitivity. The counting data were analyzed using the Chi-square (χ^2^) test. After variables associated with the primary end-point had been identified, uni- and multivariable binary logistic regression was performed. The strength of the relationship between examined variables and outcome was expressed as odds ratio (OR) with belonging 95% confidence interval (95% CI) for univariate, and as adjusted odds ratio (aOR) with belonging 95% CI for multivariate analysis. “*p*” values < 0.05 were considered significant.

## 3. Results

Seven hundred and three COVID-19 patients hospitalized in University Clinical Center Kragujevac were enrolled in the study, whose demographic and medical history characteristics are presented in Table 1. The median age of participants was 69 years, and the most common comorbidities were arterial hypertension, diabetes mellitus, and chronic kidney disease. The mortality in our cohort was 28.4% (*n =* 199). We note that non-survivors were older, had a higher prevalence of chronic kidney disease, and had higher Charlson Comorbidity Index (CCI) values than survivors. In addition, non-survivors more often required oxygen support at admission and had a shorter period between disease onset and the need for inpatient treatment.

Admission blood gas and laboratory analysis are presented in Table 2. Compared to survivors, non-survivors had significant differences in blood cell count (lower count of lymphocytes, red blood cells, hemoglobin, and platelets), had a more impaired gas exchange (lower values of partial oxygen pressure (PaO_2_) and oxygen saturation (SaO_2_)), as well as more increased laboratory markers of inflammation (including C-reactive protein (CRP), procalcitonin (PCT), lactate dehydrogenase (LDH), Interleukin-6 (IL-6), and others), kidney (including blood urea nitrogen (BUN) and serum creatinine) and myocardial injury (including high sensitive Troponin I (hsTnI), N-terminal pro-brain natriuretic peptide (NT pro-BNP), muscle brain form of creatine kinase (CKMB) and creatine kinase (CK)).

All variables that had shown a significant difference between survival categories were tested using the binary logistic regression analysis. The decision regarding the form of continuous variables in which they will be observed in the final model (continuous or categorical) was made according to the clinical relevance. The use of an accepted reference line was preferred when dividing continuous variables into categories, except for laboratory and blood gas analyses for which the great majority of patients had values below/above the reference line, in which case cut-off values had been made using the ROC analysis (Supplementary Table S1).

**Figure 1:**
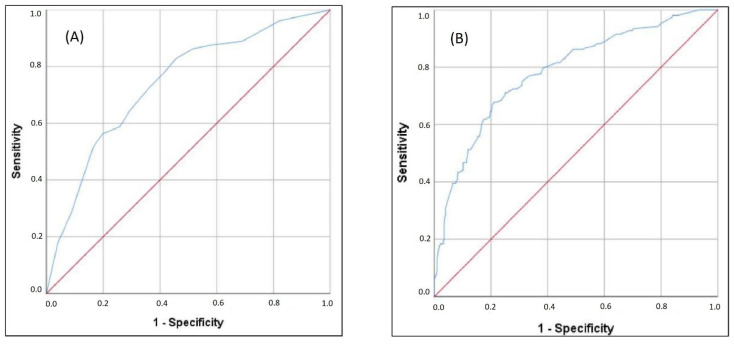
ROC analysis of (**A**) multiple regression model and (**B**) expanded multiple regression model in predicting mortality.

**Figure 2:**
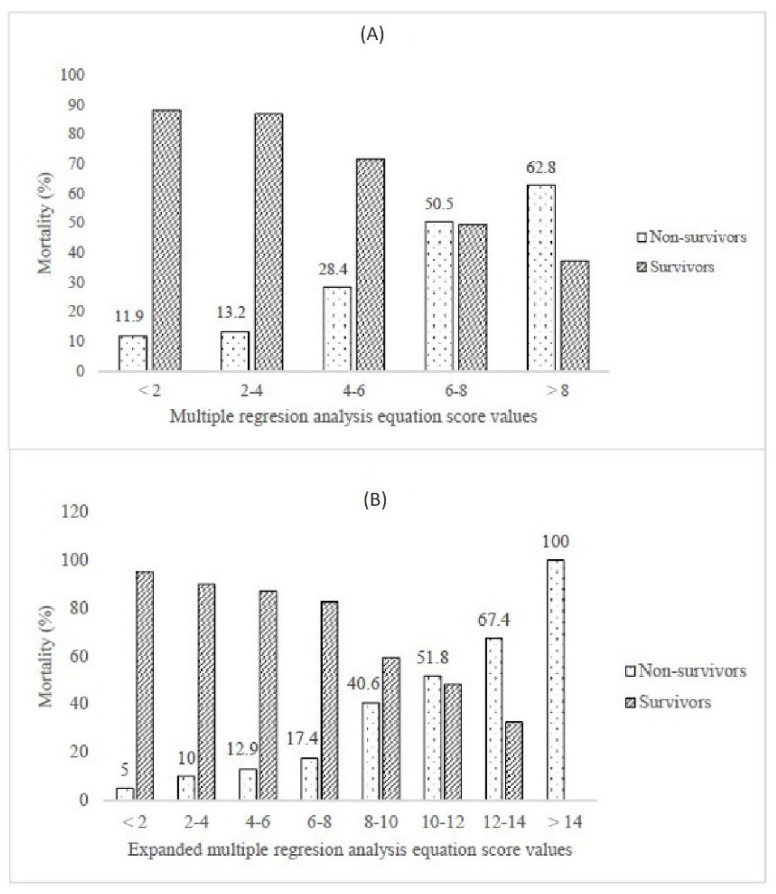
Mortality according to (**A**) multiple regression analysis and (**B**) expanded multiple regression analysis equations.

Finally, we have performed multiple binary logistic regression to examine the probability of variables to predict mortality. The model consisted of 20 variables that had been selected according to the initial univariate analysis concerning multicollinearity principles (Table 3). The model was statistically significant (c2 = 144.1, *p <* 0.001), explaining 23.7–34.5% of variance. The model had a specificity of 91.9%, a sensitivity of 39.9%, a negative predictive value of 80.5%, and a positive predictive value of 62.6%. Only four independent variables gave statistically significant contribution to the model: SaO_2_ < 88.5% (aOR 3.075), IL-6 *>* 74.6 pg/mL (aOR 2.389), LDH *>* 804.5 U/L (aOR 2.069) and age *>* 69.5 years (aOR 1.786). The logistic regression equation for predicting mortality, derived from our model is y = 0.036 + (SaO_2_ < 88.5 %) × 3.075 + (IL-6 *>* 74.6 pg/mL) × 2.389 + (LDH *>* 804.5 U/L) × 2.069 + (age *>* 69.5 years) × 1.786. The C-index of the predicted probability calculated using this multivariate logistic model was 0.740 (*p <* 0.001) (Figure 1A). The mortality according to values of the score derived from our equation is presented in Figure 2A.

We note that e-GFR < 60 mL/min, low lymphocyte count, elevated serum creatinine, and elevated hsTnI values had borderline statistical significance. The second equation expanded for these parameters is y = 0.036 + (SaO_2_ < 88.5%) × 3.075 + (IL-6 *>* 74.6 pg/mL) × 2.389 + (LDH *>* 804.5 U/L) × 2.069 + (age *>* 69.5 years) × 1.786 + (e-GFR < 60 mL/min) × 1.852 + (elevated hsTnI) × 1.765 + (low lymphocyte count) × 1.829 + (serum creatinine value > 107.5 mmol/L for males and >86.5 mmol/L for females) × 1.815. The C-index of the predicted probability calculated using this multivariate logistic model was 0.785 (*p <* 0.001) (Figure 1B). The mortality according to values of the score derived from our expanded equation is presented in Figure 2B.

## 4. Discussion

Our study provides insight into patients’ characteristics and admission laboratory findings associated with the lethal outcome of hospitalized COVID-19 patients. To our knowledge, this is the most extensive study conducted in Serbia on this topic. The cohort consisted of moderately to severely ill COVID-19 patients hospitalized between September and December 2021, in a period of the presumable predominance of delta SARS-CoV-2 variant in our country. During the research period, 1211 adult patients were hospitalized in our COVID-19 center, and 703 patients were finally enrolled in the study. We note that 508 patients were excluded, mainly due to insufficient data and incompleteness of admission laboratory parameters. The second most important reason for patient exclusion was a refusal to participate or the impossibility of obtaining a consent form (from the patient or patient’s representative) in those in a critical state upon admission or with impaired reasoning ability. Thirdly, a portion of patients initially admitted to our center had continued the inpatient treatment in other facilities after clinical stabilization. The mentioned reasons could have affected the overall cohort outcomes and admission mortality predictors.

The median time from disease onset to SARS-CoV-2 confirmation, first medical contact, and the beginning of outpatient treatment was 2.0 days. In addition, the median time between the disease confirmation and hospital admission was 5.0 days (Table 1). We note that the median times from symptoms onset to first medical contact and disease confirmation to hospital admission were shorter than reported in other publications [5,12,13]. This was mainly due to our region’s organizational structure of the COVID-19 triage algorithm—several equipped outpatient COVID-19 ambulances, available 24/7, were directly connected to the University Clinical Center Kragujevac triage posts for high-risk and moderate to severely ill patients. In addition to Serbia’s COVID-19 awareness projects, this has led to faster diagnosis confirmation, treatment initiation, and better screening for patients requiring inpatient treatment. However, despite a relatively short period until the first medical contact, the in-hospital mortality was 28.4%. In addition, a great majority of patients required oxygen support upon hospital admission (93.3%), more prominent in the non-survivors group. How the non-survivors were admitted for inpatient treatment after a shorter time from the disease onset and more frequently required oxygen support on admission, we could hypothesize that non-survivors had developed hypoxemia and disease progression faster than survivors. Thus, it would be beneficial to advance hypoxemia and disease progression screening in high-risk patients. However, timely recognition of hypoxemia in COVID-19 patients can be additionally challenged due to the absence of dyspnea and other signs of respiratory distress [21]. Half of the hospitalized patients had been admitted to ICU, and one-third had developed a critical form of the disease [3], while both events were more frequent in the non-survivors group. The most common comorbidities were arterial hypertension, diabetes, and chronic kidney disease, which is in accordance with other literature data [5,6]. However, only chronic kidney disease (CKD) and the Charlson Comorbidity Index (CCI) showed a significant difference between survival groups. Although CCI initially had OR of 1.406 with “*p*” values < 0.001, in the multivariate logistic regression model, it was not singled out as a significant predictor of mortality (Table 3). A possible explanation could be a weaker contribution to the model compared with other variables, such as age and moderate to severe CKD, as well as the fact that patients with malignant disease, as significant influencers of CCI score, were excluded from the study. Previous studies showed that CKD is a risk factor for ICU admission and unfavorable outcome [7,8,22] partially due to persistent low-grade inflammation and dysregulated immune response [23]. In addition, CKD increases the risk for acute kidney injury and the need for renal-replacement therapy, a known complication of COVID-19 [22,24]. After performing a multivariate logistic regression analysis, the initial 2.652 OR value of e-GFR, as a marker of renal function and CKD stratification, subsided to aOR of 1.852, with borderline statistical significance (Table 3). Similarly to CCI, an explanation could be a lesser contribution to the model than other variables included in the Cockroft–Gault equation, such as age and serum creatinine values.

Regarding demographic characteristics, non-survivors in our cohort were significantly older than survivors, as shown in most published papers [8,9,10,11,12]. Furthermore, after performing multivariant logistic regression, age *>* 69.5 years was an independent predictor of lethal outcome with aOR 1.786 (Table 3). Probable explanations include a higher frequency of comorbidities, such as cardiovascular diseases, diabetes, CKD, impaired immune response, and chronic inflammation associated with aging [10,25]. In our cohort, sex did not significantly impact mortality, although some published data advocate a greater mortality risk in males [10,13,14]. The mechanisms for higher mortality in men, shown in some papers, are still not fully understood. Some possible reasons are higher frequency of preexisting comorbidities, more present high-risk behavior related to COVID-19, differences in innate immune response, and different activity and expression of angiotensin-converting enzyme 2 (ACE 2) [26,27]. Therefore, the insignificant impact of sex on mortality in our cohort could be explained by different male and female representations, different patient structures (in terms of representation of age, comorbidities, and habits by sex), as well as a limited number of patients included in the study.

The degree of hypoxemia upon admission, observed through a decline in PaO_2_ and SaO_2_ values, greatly influenced an unfavorable outcome (Table 2). Furthermore, after performing multivariant logistic analysis, SaO2 below 88.5% on admission was the strongest predictor of in-hospital mortality with a three-fold risk increase (Table 3). Gas exchange impairment and the degree of hypoxemia are known risk factors for disease severity and mortality of COVID-19 patients [5,7,11,13]. Besides being a consequence of respiratory disease, hypoxemia can further contribute to lung tissue damage by enhancing various cytotoxic functions and promoting inflammation [5,28].

Regarding laboratory findings on admission, sixteen parameters initially showed an impact on mortality, including differences in blood cell count, increased laboratory markers of inflammation, and renal and myocardial injury (Table 2 and Table 3). However, after performing multivariant logistic regression analysis, only IL-6 (aOR 2.389 for values higher than 74.6 pg/mL) and LDH (aOR 2.069 for values higher than 804.5 U/L) remained independent predictors of in-hospital mortality (Table 3). These parameters are one of the most commonly advocated COVID-19 severity and mortality predictors, suggesting a significant inflammatory component in disease deterioration. Though, considerable variations of the proposed cut-off and aOR values are seen across the literature [8,10,12,13,15,16,17,18]. Regarding other inflammatory biomarkers, we note that CRP and PCT significantly differ between survival groups, which further supports the vital inflammatory component of the disease progression. However, they were not singled out as independent mortality predictors in the multivariant regression analysis, possibly due to a weaker contribution to the regression model than other variables.

We note that cardiac troponin I, lymphocyte count, and serum creatinine values, all cited as mortality predictors in the literature [8,10,12,13,15,16,17,18,29,30], had statistical significance levels between 0.05 and 0.09 in the final regression model, and therefore should not be neglected [31] (Table 3). Acute myocardial injury, observed as an elevated troponin values, is a recognized COVID-19 complication that significantly impacts the further course of the disease and whose underlying mechanisms are complex and still not fully understood [7,10,15,16,17,18,29,30]. Similarly, elevated serum creatinine levels, suggestive of kidney injury, have been associated with a worse outcome in hospitalized COVID-19 patients [9,10,15,17,18,23]. Several mechanisms of acute kidney injury in COVID-19 have been proposed [32]. However, due to study limitations in terms of the absence of insight into renal function before the disease onset and trends of creatinine values during the hospitalization, high creatinine values in our cohort cannot be linked with acute kidney injury alone. Nevertheless, elevated creatinine values on admission should be considered when estimating the mortality risk.

Finally, after performing multivariant logistic regression, our study provided a mortality risk assessment score with satisfactory efficiency based on the data available in the first hours of hospital admission. The initial model, which included SaO_2_ < 88.5%, IL-6 *>* 74.6 pg/mL, LDH *>* 804.5 U/L, and age *>* 69.5 years, based on a significance level of <0.05, had a C-index of 0.740 (Figure 1A and Figure 2A). However, implementing additional variables, based on a significance level of <0.09 (e-GFR, hsTnI, lymphocyte count, and serum creatinine), the C-index reached a maximum value of 0.785 (Figure 1B and Figure 2B).

More than thirty different predictors of COVID-19 mortality can be found in the available literature, including sociodemographic and medical history data, the extent of lung involvement on imaging, blood exchange impairment, and various laboratory abnormalities [5,6,7,8,9,10,11,12,13,14,15,16,17,18]. However, the selection of significant predictors, their cut-off, and aOR values differs across the literature. These variations can be explained through: differences in methodological and statistical approach, variables selection and availability, cohort characteristics (sociodemographic characteristics, comorbidities burden, representation of different severity forms of the disease, and others), time of laboratory analysis sampling, a predominance of different SARS-CoV-2 variants, and others. Therefore, some differences in mortality risk assessment models are expected, and no published model could be used with complete reliability in all the settings, implying different geographical, sociodemographic, hospital equipment, patient structure, and other variable conditions. Instead, such findings support the assertion that variables available on hospital admission can be valuable in predicting the mortality risk of COVID-19 patients and encourage physicians to further studies on this topic.

## 5. Conclusions

Compared to survivors, hospitalized COVID-19 non-survivors significantly differ in sociodemographic characteristics, comorbidities, blood gas, and laboratory analysis. In addition, these data, available in the first hours upon hospital admission, can help assess the mortality risk of hospitalized COVID-19 patients.

## 6. Study Limitations

Certain limitations are present in this study. Firstly, data were acquired during the emergency condition, and thus the completeness of data recording was less than optimal, especially during hospital admission. This limitation particularly applies to the number of patients with complete laboratory analyses of interest, which greatly influenced the number of patients finally included in the study. Secondly, the number of laboratory analyses included in the research is limited by our central laboratory equipment. However, the primary study goal was to single out significant admission predictors of COVID-19 mortality based on the parameters available in most hospitals, including our Clinical Center. Thirdly, thoracic computed tomography (CT), as a radiographic method of the highest accuracy, had not been included in our mortality risk assessment on admission. Although the published data recognizes the importance of CT imaging in predicting COVID-19 mortality [33,34], a limited number of patients underwent CT diagnostics upon admission, so it could not be included in further analysis.

## Figures and Tables

**Table 1:** Demographic and medical history characteristics of COVID-19 survivors and non-survivors.

Cohort Characteristics		Frequency (Number of Cases) or Median Value (with IQR)			
		Cohort	Survivors	Non-Survivors	*p* Value
Age		69.0 (IQR 17.0)	67.0 (IQR 19.0)	73.0 (IQR 13.0)	<0.001 *
Gender	Male	57.9% (*n =* 407)	73.1% (*n =* 297)	26.9% (*n =* 110)	0.353
	Female	42.1% (*n =* 296)	69.6% (*n =* 206)	30.4% (*n =* 90)	
**Comorbidities**					
Arterial hypertension		68.2% (*n =* 477)	66.1% (*n =* 331)	73.7% (*n =* 146)	0.061
Diabetes mellitus		28.1% (*n =* 197)	26.7% (*n =* 134)	31.8% (*n =* 63)	0.211
Chronic kidney disease		22.9% (*n =* 161)	17.7% (*n =* 89)	36.4% (*n =* 72)	<0.001 *
Neurological condition ^1^		9.6% (*n =* 67)	8% (*n =* 40)	13.6% (*n =* 27)	0.032 *
Atrial fibrillation		7.6% (*n =* 53)	7% (*n =* 35)	9.1% (*n =* 18)	0.420
Previous myocardial infarction		6.4% (*n =* 45)	6.8% (*n =* 34)	5.6% (*n =* 11)	0.681
Malignancy		6.3% (*n =* 44)	6.4% (*n =* 32)	6.1% (*n =* 12)	1.000
Obstructive lung disease ^2^		4.6% (*n =* 32)	4% (*n =* 20)	6% (*n =* 12)	0.332
Charlson Comorbidity Index		3.0 (IQR 2.0)	3.0 (IQR 2.0)	4.0 (IQR 2.0)	<0.001 *
**Disease Course and Outcome**					
Days from disease onset to hospital admission		7.0 (IQR 5.0)	7.0 (IQR 5.0)	6.0 (IQR 4.0)	<0.001 *
Days from SARS-CoV-2 verification to hospital admission		5.0 (IQR 6.0)	6.0 (IQR 6.0)	4.0 (IQR 6.0)	<0.001 *
Critical form development		33.7% (*n =* 236)	20.5%	66.8%	<0.001 *
ICU admission		50.1% (*n =* 351)	34.5% (*n =* 243)	89.4% (*n =* 628)	<0.001 *
Hospital stay (days)		14.0 (IQR 10.0)	15.0 (IQR 12.0)	12.0 (IQR 10.0)	<0.001 *
Oxygen support requirement on admission		93.3% (*n =* 656)	91.8% (*n =* 645)	97.0% (*n =* 682)	0.022 *

Abbreviations: ICU—intensive care unit; IQR—interquartile range. *—statistical significance level at <0.5. ^1^ Neurological condition: the presence of either history of stroke, brain tumor or malformation, vascular disease, dementia of any etiology, etc. ^2^ Obstructive lung disease: the presence of either chronic obstructive lung disease or bronchial asthma.

**Table 2:** Admission blood gas and laboratory analysis of COVID-19 survivors and non-survivors.

Laboratory Analysis	Median Values (IQR)			
	Cohort	Survivors	Nonsurvivors	*p* Value
PaO_2_ [kPa]	7.1 (IQR 1.4)	7.2 (IQR 1.5)	6.7 (IQR 1.5)	<0.001 *
SaO_2_ [%]	89 (IQR 7)	90 (IQR 6)	87 (IQR 9)	<0.001 *
WBC [10^9^/L]	7.84 (IQR 4.7)	7.94 (IQR 4.7)	7.66 (IQR 4.9)	0.198
Gran [10^9^/L]	6.3 (IQR 4.4)	6.30 (IQR 4.39)	6.30 (IQR 4.89)	0.560
Lym [10^9^/L]	0.7 (IQR 0.5)	0.73 (IQR 0.5)	0.68 (IQR 0.43)	0.001 *
RBC [10^12^/L]	4.5 (IQR 0.77)	4.52 (IQR 0.76)	4.34 (IQR 0.73)	<0.001 *
HGB [g/L]	133 (IQR 3)	134 (IQR 11)	129 (IQR 25)	0.002 *
PLT [10^9^/L]	195 (IQR 103)	205 (IQR 108)	170 (IQR 83)	<0.001 *
INR	1.07 (IQR 0.19)	1.07 (IQR 0.19)	1.09 (IQR 0.2)	0.252
aPTT [s]	31.95 (IQR 7.67)	31.4 (IQR 7.7)	33.1 (IQR 8.0)	0.004 *
Fibrinogen [g/L]	6.25 (IQR 2.0)	6.26 (IQR 2.06)	6.22 (IQR 2.09)	0.219
DD [ug/mL]	0.99 (IQR 1.25)	0.94 (IQR 1.14)	1.09 (IQR 1.51)	0.058
Albumin [g/L]	36 (IQR 5)	36 (IQR 6)	35 (IQR 5)	0.055
AST [IU/L]	42 (IQR 34)	42 (IQR 34)	42 (IQR 38)	0.721
ALT [IU/L]	35 (IQR 35)	37 (IQR 37)	31 (IQR 31)	0.001 *
GGT [IU/L]	40 (IQR 57.25)	41 (IQR 56.5)	37 (IQR 56)	0.139
BUN [mmol/L]	7.9 (IQR 5.6)	7.5 (IQR 5.2)	9.2 (IQR 7.4)	<0.001 *
Creatinine [mmol/L]	95 (IQR 47)	90.5 (IQR 41)	109 (IQR 77)	<0.001 *
LDH [U/L]	784 (IQR 365.5)	756 (IQR 355.25)	889 (IQR 416)	<0.001 *
Ferritin [ug/L]	809 (IQR 965)	798 (IQR 936)	840 (IQR 1129)	0.397
CK [U/L]	114 (IQR 190)	105 (IQR 170)	150 (IQR 259.5)	0.002 *
CKMB [U/L]	20 (IQR 11)	19 (IQR 11)	22 (IQR 12)	<0.001 *
CRP [mg/L]	104 (IQR 90.5)	99.1 (IQR 88.5)	109.1 (IQR 103.5)	0.013 *
PCT [ng/mL]	0.116 (IQR 0.18)	0.1 (IQR 0.14)	0.17 (IQR 0.342)	<0.001 *
hsTnI [ng/mL]	0.006 (IQR 0.019)	0.001 (IQR 0.012)	0.016 (IQR 0.042)	<0.001 *
pro-BNP [pg/mL]	667 (IQR 1394)	580 (IQR 976)	1102 (IQR 3426)	<0.001 *
IL-6 [pg/mL]	67.85 (IQR 90.13)	56.5 (IQR 79.15)	98.1 (IQR 103.2)	<0.001 *

Abbreviations: ALT—alanine transaminase; aPTT—activated partial thromboplastin clotting time; AST—aspartate transaminase; BUN—blood urea nitrogen; CK—creatine kinase; CKMB—muscle-brain form of creatine kinase; CRP—c reactive protein; DD- D dimer; GGT—gamma-glutamyl transferase; Gran—granulocytes; Hgb—hemoglobin; hsTnI—high sensitive troponin I; IL-6—interleukin 6; INR—international normalized ratio; LDH—lactate dehydrogenase; Lym—lymphocytes; NT pro-BNP—N-terminal pro-brain natriuretic peptide; PaO_2_—Partial pressure of oxygen; PCT—procalcitonin; PLT—platelets; RBC—red blood cells; SaO_2_—oxygen saturation of blood; WBC—white blood cells; *—statistical significance level at <0.5.

**Table 3:** Crude and adjusted OR values for variables available on hospital admission in regard to predicting in-hospital mortality of COVID-19 patients.

Variable		Frequency of Mortality	Crude OR		Adjusted OR	
			OR (95% CI)	*p* Value	aOR (95% CI)	*p* Value
Age [years]	<69.5	18.2%	1	/	1	/
	>69.5	39.5%	2.928 (2.075–4.131)	<0.001 *	1.786 (1.017–3.138)	0.044 *
PaO_2_ [kPa]	≥6.75	22%	1	/	Excluded for multicollinearity **	
	<6.75	38.3%	2.200 (1.563–3.097)	<0.001 *		
SaO_2_ [%]	≥88.5	21.8%	1	/	1	/
	<88.5	38.5%	2.242 (1.595–3.151)	<0.001 *	3.075 (1.919–4.928)	<0.001 *
Lym [10^9^/L]	≥1.2 × 10^9^ /L	20.5%	1	/	1	/
	<1.2 × 10^9^ /L	29.7%	1.638 (1.002–2.678)	0.049 *	1.829 (0.914–3.661)	0.088
PLT [10^9^/L]	≥135	25.8%	1	/	1	/
	<135	43.3%	2.189 (1.425–3.362)	<0.001 *	1.605 (0.886–2.908)	0.118
HGB [g/L]	Male ≥ 138 Female ≥ 120	24.8%	1	/	1	/
	Male < 138 Female < 120	38.9%	1.934 (1.350–2.771)	<0.001 *	1.318 (0.784–2.214)	0.298
ALT [IU/L]	≥41	23.9%	1	/	1	/
	<41	31.7%	1.475 (1.050–2.072)	0.025 *	0.954 (0.579–1.571)	0.853
BUN [mmol/L]	<7.75	20.1%	1	/	1	/
	≥7.75	35.8%	2.212 (0.573–3.125)	<0.001 *	0.797 (0.437–1.456)	0.461
Creatinine [mmol/L]	Male < 107.5 Female < 86.5	20.6%	1	/	1	/
	Male ≥ 107.5 Female ≥ 86.5	37.8%	2.312 (1.650–3.239)	<0.001 *	1.815 (0.947–3.478)	0.073
LDH [U/L]	<804.5	21.1%	1	/	1	/
	≥804.5	36.2%	2.126 (1.483–3.049)	<0.001 *	2.069 (1.233–3.472)	0.006 *
CKMB [U/L]	≤25	24.5%	1	/	1	/
	>25	36.3%	1.775 (1.202–2.560)	0.004 *	0.689 (0.392–1.210)	0.194
CK [U/L]	≤171	24.4%	1	/	1	/
	>171	35.3%	1.691 (1.208–2.368)	0.002 *	0.996 (0.591–1.678)	0.988
CRP [mg/L]	<107.5	25.4%	1	/	1	/
	≥107.5	31.6%	1.359 (0.978–1.889)	0.068	1.245 (0.748–2.071)	0.399
PCT [ng/mL]	<0.129	20.7%	1	/	1	/
	≥0.129	36.4%	2.195 (1.562–3.085)	<0.001 *	0.716 (0.419–1.222)	0.221
hsTnI [ng/mL]	Males < 0.0342 Females < 0.0156	21.6%	1	/	1	/
	Males ≥ 0.0342 Females ≥ 0.0156	53%	4.081 (2.795–5.959)	<0.001 *	1.765 (0.978–3.184)	0.059
NT pro-BNP [pg/mL]	<759	20.6%	1	/	1	/
	≥759	37.4%	2.299 (1.639–3.224)	<0.001 *	0.808 (0.471–1.385)	0.438
IL-6 [pg/mL]	<74.6	18.7%	1	/	1	/
	≥74.6	38.8%	2.761 (1.942–3.926)	<0.001 *	2.389 (1.442–3.957)	0.001 *
Days from symptom onset	/	/	0.899 (0.857–0.944)	<0.001 *	0.925 (0.824–1.037)	0.181
Days from disease confirmation	/	/	0.901 (0.860–0.945)	<0.001 *	Excluded for multicollinearity **	
CCI	/	/	1.406 (1.277–1.549)	<0.001 *	1.138 (0.951–1.361)	0.157
Neurological condition	No	27.1%	1	/	1	/
	Yes	40.3%	1.820 (1.083–3.057)	0.024 *	1.289 (0.628–2.645)	0.488
e-GFR	C-G ≥ 60	23.4%	1	/	1	/
	C-G < 60	44.7%	2.652 (1.833–3.836)	<0.001 *	1.852 (0.941–3.646)	0.075

Abbreviations: ALT—alanine transaminase; aOR—adjusted OR; BUN—blood urea nitrogen; CCI—Charlson comorbidity index; C-G—Cocroft-Gault formula for estimating glomerular filtration rate; CI—confidence interval; CK—creatine kinase; CKMB—muscle–brain form of creatine kinase; CRP—c reactive protein; e-GFR—estimated glomerular filtration rate; Hgb—hemoglobin; hsTnI—high sensitive troponin I; IL-6—interleukin 6; LDH—lactate dehydrogenase; Lym—lymphocytes; NT pro-BNP—N-terminal pro-brain natriuretic peptide; OR—odds ratio; PaO_2_—Partial pressure of oxygen; PCT—procalcitonin; PLT—platelets; SaO_2_—oxygen saturation of the blood. *—statistical significance level at <0.5. **—variables excluded due to the multicollinearity principle of multiple logistic regression.

## Data Availability

The data presented in this study are available on request from the corresponding author.

## Supplementary Materials

The following supporting information can be downloaded at: https://www.mdpi.com/article/10.3390/jcm11206109/s1, Table S1: Threshold values for blood gas and laboratory parameters, according to the ROC analysis or laboratory reference lines.
