# Supplementary material for: Admission Predictors of Mortality in Hospitalized COVID-19 Patients—A Serbian Cohort Study
Source: J Clin Med. 2022 Oct 17;11(20):6109. doi: 10.3390/jcm11206109 (PMC9605560; doi:10.3390/jcm11206109)
Supplement: Supplementary file 1 [file jcm-11-06109-s001.zip › jcm-1945793-supplementary.pdf]

Supplementary Table S1. Threshold values for blood gas and laboratory parameters, according to the ROC analysis or laboratory reference lines.

Parameter	Threshold Value	AUC (CI)	„*p*“ value
SaO2 [%]	88.5	0.597 (0.549-0.645)	< 0.001*
PaO2 [kPa]	6.75	0.596 (0.547-0.644)	< 0.001*
Lymphocytes [10^9/L]	1.2	*Laboratory reference line*	
PLT [10^9/L]	135	*Laboratory reference line*	
HGB [g/L]	Male:138<br>Female:120	*Laboratory reference line*	
ALT [IU/L]	41	*Laboratory reference line*	
BUN [mmol/L]	7.75	0.596 (0.550-0.642)	< 0.001*
Creatinin [mmol/L]	Male: 107.5<br>Female: 86.5	0.603 (0.557-0.650)	< 0.001*
LDH [U/L]	804.5	0.593 (0.543-0.643)	< 0.001*
CKMB [U/L]	25	*Laboratory reference line*	
CK [U/L]	171	*Laboratory reference line*	
CRP [mg/L]	107.5	0.560 (0.513-0.608)	0.013*
PCT [ng/mL]	0.129	0.597 (0.550-0.644)	< 0.001*
hsTnI	Males: 0.0342<br>Females: 0.0156	*Laboratory reference line*	

NT pro-BNP [pg/mL]	759	0.603 (0.556-0.649)	< 0.001*
IL-6 [pg/mL]	74.6	0.624 (0.577-0.671)	< 0.001*

Abbreviations: ALT - alanine transaminase; AUC - area under the curve; BUN - blood urea nitrogen; CI - confidence interval; CK - creatine kinase; CKMB - muscle-brain form of creatine kinase; CRP - c reactive protein; HGB - hemoglobin; ; hsTnI - high sensitive troponin I; IL-6 - interleukin 6; LDH - lactate dehydrogenase; NPV - negative predictive value; PaO2 - Partial pressure of oxygen; PCT - procalcitonin; PLT - platelets; PPV - positive predictive value; pro-BNP - a pro-form of brain natriuretic peptide; ROC - receiver operating characteristics curve; SaO2 - oxygen saturation of blood; * statistical significance level at < 0.5.
